# TRIM59在非小细胞肺癌中的表达及其与预后的关系

**DOI:** 10.3779/j.issn.1009-3419.2020.01.04

**Published:** 2020-01-20

**Authors:** 海英 田, 东旸 张, 荣建 徐, 毅 秦, 亚良 兰, 文捷 矫, 玉栋 韩

**Affiliations:** 1 266000 青岛，青岛大学附属医院肿瘤放疗科 Department of Radiation Oncology, Affiliated Hospital of Qingdao University, Qingdao 266000, China; 2 266000 青岛，青岛大学附属医院胸外科 Department of Thoracic Surgery, Affiliated Hospital of Qingdao University, Qingdao 266000, China

**Keywords:** TRIM59, 肺肿瘤, 预后, TRIM59, Lung neoplasms, Prognosis

## Abstract

**背景与目的:**

TRIM家族蛋白是E3泛素连接酶的重要成员，诸多研究证实TRIM家族成员在多种肿瘤的发生发展中发挥了重要作用。前期我们通过高通量测序发现TRIM59 mRNA在非小细胞肺癌组织中表达明显升高。本研究旨在探讨TRIM59在非小细胞肺癌中的表达变化及其与患者临床病理特征和预后的关系。

**方法:**

检索高通量芯片表达谱数据库（Gene Expression Omnibus, GEO）和肿瘤基因组计划（The Cancer Genome Atlas, TCGA）数据库中的非小细胞肺癌基因表达谱数据和临床数据，分析TRIM59 mRNA的表达与非小细胞肺癌患者预后的关系；采用免疫组织化学染色的方法检测TRIM59蛋白在90例肺癌组织及癌旁组织中表达，分析TRIM59蛋白的表达与患者临床病理参数及预后的关系。

**结果:**

TRIM59 mRNA在肺癌组织中表达明显升高并提示预后不良；TRIM59蛋白在肺癌组织中高表达，在癌旁组织低表达或不表达，TRIM59蛋白的表达与肿瘤大小（*P*=0.007）、病理分级（*P*=0.009）、肿瘤原发灶-淋巴结-转移（tumor-node-metastasis, TNM）分期（*P*=0.003）和淋巴结转移（*P*=0.003）密切相关。TRIM59蛋白高表达的患者预后较差，*Cox*多因素回归分析发现TRIM59蛋白的表达和TNM分期是影响患者预后的独立危险因素。

**结论:**

TRIM59的表达水平与非小细胞肺癌的预后密切相关，是影响患者预后的独立危险因素。

## Abstract (en)

**Background and objective:**

TRIM proteins are important members of E3 ubiquitin ligases, and many studies have confirmed that TRIM family members play an important role in the development of various tumors. We found that TRIM59 expression level in non-small cell lung cancer (NSCLC) was significantly increased through second-generation sequencing. The purpose of this study was to investigate the expression of TRIM59 in NSCLC and its relationship with the clinicopathological parameters as well as the prognosis of patients.

**Methods:**

The Cancer Genome Atlas (TCGA) and Gene Expression Omnibus (GEO) datasets were excavated to analyze the expression of TRIM59 mRNA in NSCLC and its relationship with the prognosis of patients; The expression of TRIM59 protein in 90 tumor tissues and adjacent tissues was detected by immunohistochemical staining, and the relationship between the expression of TRIM59 protein and clinicopathological parameters and prognosis was analyzed.

**Results:**

Overexpression of TRIM59 mRNA in tumor tissues predicted poor prognosis. The expression level of TRIM59 protein was significantly higher in tumor tissues than in adjacent tissues, and TRIM59 protein expression was correlated with tumor size (*P*=0.007), tumor differentiation (*P*=0.009), tumor-node-metastasis (TNM) stage (*P*=0.003) and lymph node metastasis (*P*=0.003). Multivariate *Cox* regression analyses showed that along with TNM stage, overexpression of TRIM59 could be considered an independent prognostic factor for NSCLC patients.

**Conclusion:**

The expression of TRIM59 is closely related to the prognosis of NSCLC patients, and it is an independent risk factor for NSCLC patients.

肺癌是发病率和死亡率高居第一位的恶性肿瘤，对人类的身体健康构成了极大的威胁，其中大部分是非小细胞肺癌（non-small cell lung cancer, NSCLC）^[1]^。肺癌的发生发展与基因表达及信号通路传导异常密切相关，研究该过程中的具体分子机制是当今NSCLC基础研究的热点^[2]^。我们前期通过高通量测序技术发现TRIM59 mRNA在肺癌组织中的表达量高于癌旁组织。TRIM59已被报道与脑胶质瘤^[3]^、胃癌^[4]^、胆管癌^[5]^、乳腺癌^[6, 7]^、卵巢癌^[8]^等多种癌症密切相关，但与NSCLC的关系尚不清楚。在该研究中，首先通过挖掘公共数据库中的NSCLC数据信息，分析TRIM59 mRNA在NSCLC中的表达及其与预后的关系；进一步通过检测TRIM59蛋白在NSCLC中的表达水平，分析其与临床病理特征及患者预后的关系，为NSCLC的诊断和治疗提供一定的思路。

## 1 材料与方法

### 1.1 高通量芯片表达谱数据库（Gene Expression Omnibus, GEO）和肿瘤基因组计划（The Cancer Genome Atlas, TCGA）数据库检索

本研究中通过检索TCGA（http://cancergenome.nih.gov/）数据库和GEO（ttps://www.ncbi.nlm.nih.gov/geo/）数据库中的NSCLC基因表达谱数据和临床数据，用于基因差异分析和预后分析。

### 1.2 研究对象及临床资料

收集2009年1月-2010年12月在青岛大学附属医院胸外科进行手术切除的90例患者的NSCLC组织和癌旁组织标本。入组标准：初诊，术前未放化疗，术后病理为NSCLC，围术期未出现严重的并发症，所有病例的临床资料、病理分型分期及随访信息完整，随访截止时间2018年12月。该研究经青岛大学附属医院伦理委员会批准，并取得患者及家属的知情同意。

### 1.3 免疫组织化学方法检测TRIM59蛋白在肺癌及癌旁组织中的表达

#### 1.3.1 免疫组织化学试剂

一抗为TRIM59多克隆抗体（货号：HPA017750，Sigma公司，美国），工作浓度是1:150；SP法免疫组化试剂盒：3% H_2_O_2_，封闭用正常血清工作液，生物素化通用二抗工作液，辣根酶标记链酶卵白素工作液（中杉金桥，中国）；DAB显色试剂盒（Invitrogen公司，美国）；苏木素染料（中杉金桥，中国）。

#### 1.3.2 免疫组织化学步骤

（1）脱蜡：玻片置于60 ℃烤箱中2.5 h；二甲苯脱蜡15 min，共2次；梯度酒精水化5 min，共4次；ddH_2_O中浸泡5 min。（2）抗原修复：玻片置于抗原修复液中，微波炉中高火加热3 min（91 ℃-93 ℃），室温静置5 min（77 ℃-78 ℃），低火加热24 min（80 ℃-90 ℃）。（3）去内源性过氧化物酶：加3% H_2_O_2_，孵育5 min，PBS冲洗3 min，共3次。（4）封闭：滴加适量的Blocking Solution（BS），孵育10 min；吸弃BS，不冲洗。（5）一抗孵育：滴加适量的一抗（3% BSA-PBS稀释），Parafilm膜覆盖后置于湿盒内4 ℃过夜。（6）二抗孵育：玻片室温孵育30 min，PBS清洗3 min，3次；滴加适量的生物素化通用二抗，室温孵育15 min；PBS清洗3 min，3次；滴加适量的辣根酶标记链酶卵白素工作液，室温孵育15 min；PBS清洗3 min，3次。（7）DAB染色：滴加适量的DAB稀释液，染色，ddH_2_O终止反应。（8）苏木素复染：苏木素染色6 s，清水漂洗，PBS内反蓝10 min。（9）脱水封片：梯度酒精5 min，4次，二甲苯5 min，2次，中性树胶封片。

#### 1.3.3 免疫组织化学评定方法

由两位经验丰富的病理科医师对切片进行双盲阅片。根据细胞染色强度评分和阳性细胞比例评分相加进行半定量分析。根据阳性细胞染色强度评分：0分（无细胞染色），1分（浅棕色颗粒），2分（棕黄色颗粒），3分（褐色颗粒）。根据阳性细胞所占比例评分：0分（阳性细胞数少于10%），1分（阳性细胞数介于10%-25%），2分（阳性细胞数介于26%-50%），3分（阳性细胞数在50%以上）。将两项得分相加即为最后得分，分为两组：0分-2分为阴性表达，3分-6分为阳性表达。

### 1.4 统计分析

采用SPSS 16.0软件（Chicago, IL, USA）对数据进行统计分析，采用GraphPad Prism 7（San Diego, CA, USA）软件对数据进行作图，采用Image J（National Institutes of Health）软件分析免疫组织化学染色结果。用均数±标准差（Mean±SD）表示计量资料，统计分析采用*t*检验，单因素分析采用*χ*^2^检验。生存分析采用*Kaplan-Meier*生存曲线和*Cox*回归分析。*P* < 0.05为差异具有统计学意义。

## 2 结果

### 2.1 TRIM59 mRNA在NSCLC组织中的表达高于癌旁组织

前期利用高通量测序技术发现TRIM59 mRNA在NSCLC组织中的表达明显高于癌旁组织（图 1A，*P*=0.000, 5，配对*t*检验）。通过挖掘GEO数据库和TCGA数据库验证了此发现：GSE19804数据集包含60对NSCLC组织及对应的癌旁组织（图 1B，*P* < 0.000, 1），GSE19188数据集包含91例肺癌组织和65例癌旁组织（图 1C，*P* < 0.000, 1），TCGA肺腺癌数据集包含513例肺腺癌组织和58例癌旁组织（图 1D，*P* < 0.000, 1），TCGA肺鳞癌数据集包含502例肺鳞癌组织和51例癌旁组织（图 1E，*P* < 0.000, 1），结果均显示肺癌组织中TRIM59 mRNA的表达量显著高于癌旁组织。同时发现随着肿瘤分期升高，TRIM59 mRNA的表达量也随之升高（图 1F），上述结果说明TRIM59与NSCLC的发生发展有一定关系。

**1:**
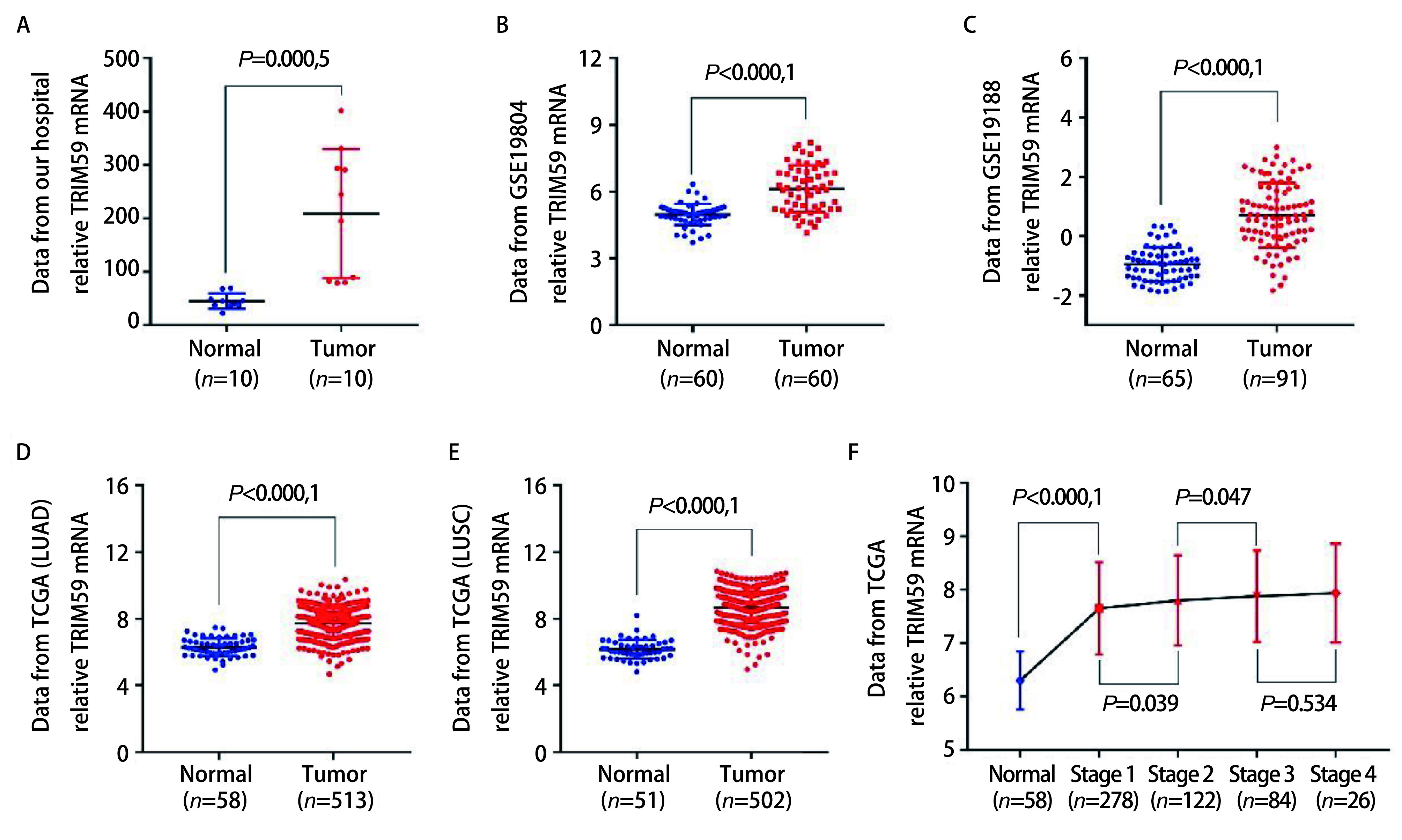
TRIM59 mRNA在NSCLC组织和癌旁组织的表达。A-E：TRIM59 mRNA在NSCLC组织中的表达明显高于癌旁组织（A：高通量测序数据；B：来自GSE19804数据集；C：来自GSE19188数据集；D：来自TCGA肺腺癌数据集；E：来自TCGA肺鳞癌数据集）；F：随着肿瘤分期的增高，TRIM59 mRNA的表达量有增高趋势 The expression level of TRIM59 mRNA in tumor and normal adjacent tissues. A-E: The expression level of TRIM59 mRNA was higher in tumor tissues compared with adjacent tissues (A: Data from our hospital; B: Data from GSE19804; C: Data from GSE19188; D: Data from TCGA lung adenocarcinoma; E: Data from TCGA lung squamous cell carcinoma); F: The expression level of TRIM59 mRNA is higher in advanced stage. GEO: Gene Expression Omnibus; TCGA: The Cancer Genome Atlas. NSCLC: non-small cell lung cancer

### 2.2 TRIM59 mRNA的表达水平与NSCLC患者预后的关系

为了深入了解TRIM59的表达与患者预后之间的关系，我们分析了GSE31210和GSE30219数据集，以中位数作为cutoff值，根据TRIM59的表达量分为高表达组和低表达组，不论是无复发生存期还是总生存期，TRIM59高表达的患者均低于TRIM59低表达患者（图 2A-图 2D）。同时我们也分析了TCGA数据库中NSCLC患者的无复发生存期和总生存期，得到了同样的结果（图 2E、图 2F）。上述数据进一步说明TRIM59在NSCLC的发生和进展过程中发挥了重要作用。

**2:**
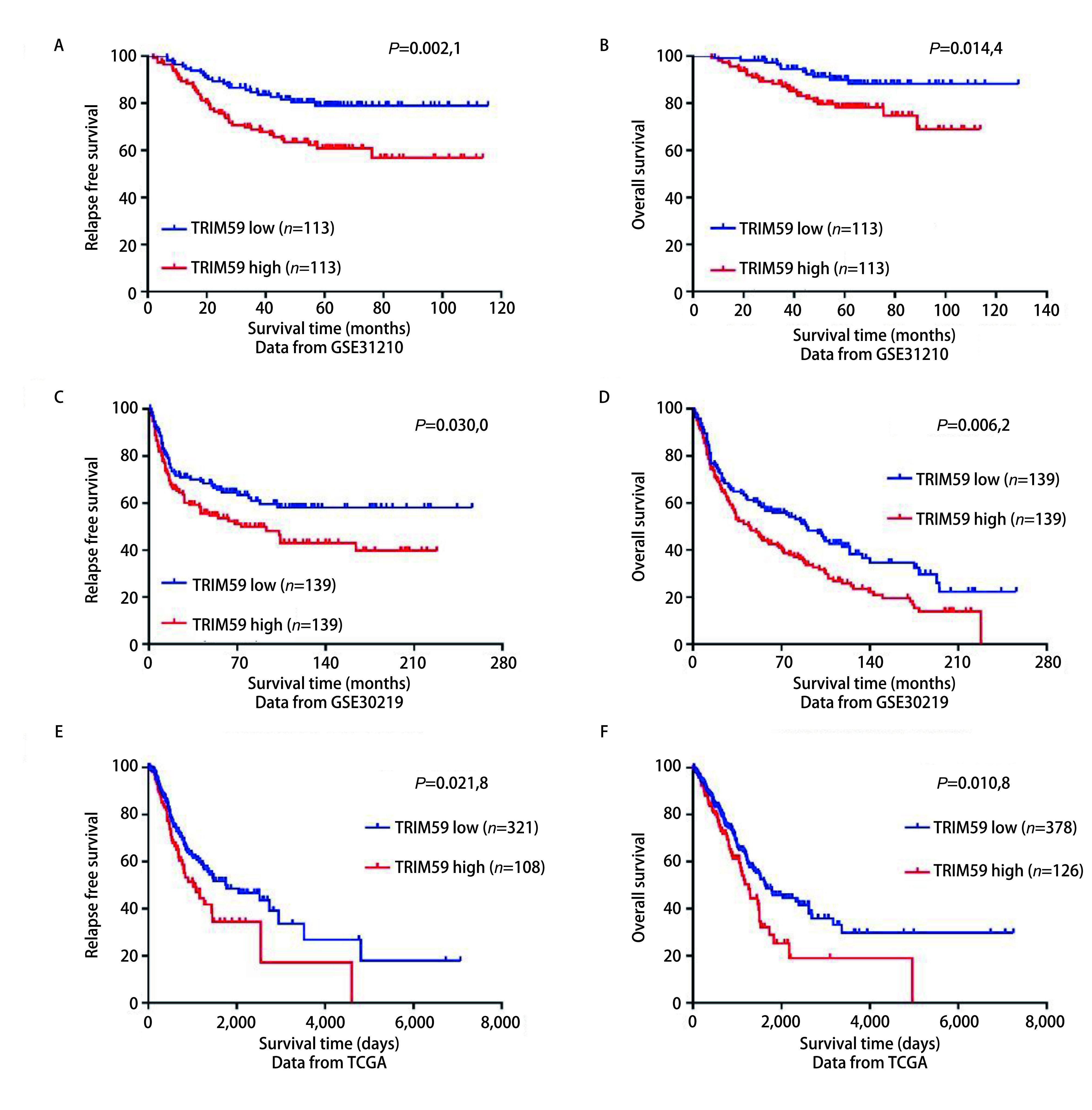
TRIM59 mRNA高表达与NSCLC患者预后不良相关。A-B：GSE31210数据集患者的生存分析；C-D：GSE30219数据集患者的生存分析；E-F：TCGA数据集患者的生存分析 TRIM59 mRNA overexpression was associated with poor prognosis in NSCLC. A-B: Survival analysis of NSCLC patients from GSE31210 dataset; C-D: Survival analysis of NSCLC patients from GSE30219 dataset; E-F: *Kaplan*-*Meier* survival analysis of NSCLC patients from TCGA dataset

### 2.3 TRIM59蛋白表达与临床病理特征的关系

利用免疫组化方法检测TRIM59蛋白在90对NSCLC组织和癌旁组织中的表达，结果发现TRIM59在癌组织中高表达，在癌旁组织低表达或不表达（图 3A），两组之间比较有显著的统计学差异（图 3B，*P* < 0.000, 1），其中有52例TRIM59呈阳性表达，38例TRIM59呈阴性表达。TRIM59定位于胞浆或核，阳性表达呈弥漫染色，淡黄至棕黄、棕褐色。我们分析了TRIM59的表达与临床病理特征的关系，发现TRIM59的表达与肿瘤大小(*P*=0.007）、TNM分期(*P*=0.003）、淋巴结转移(*P*=0.003）、分化程度(*P*=0.009）密切相关，与年龄、性别、病理类型无关（表 1）。

**3:**
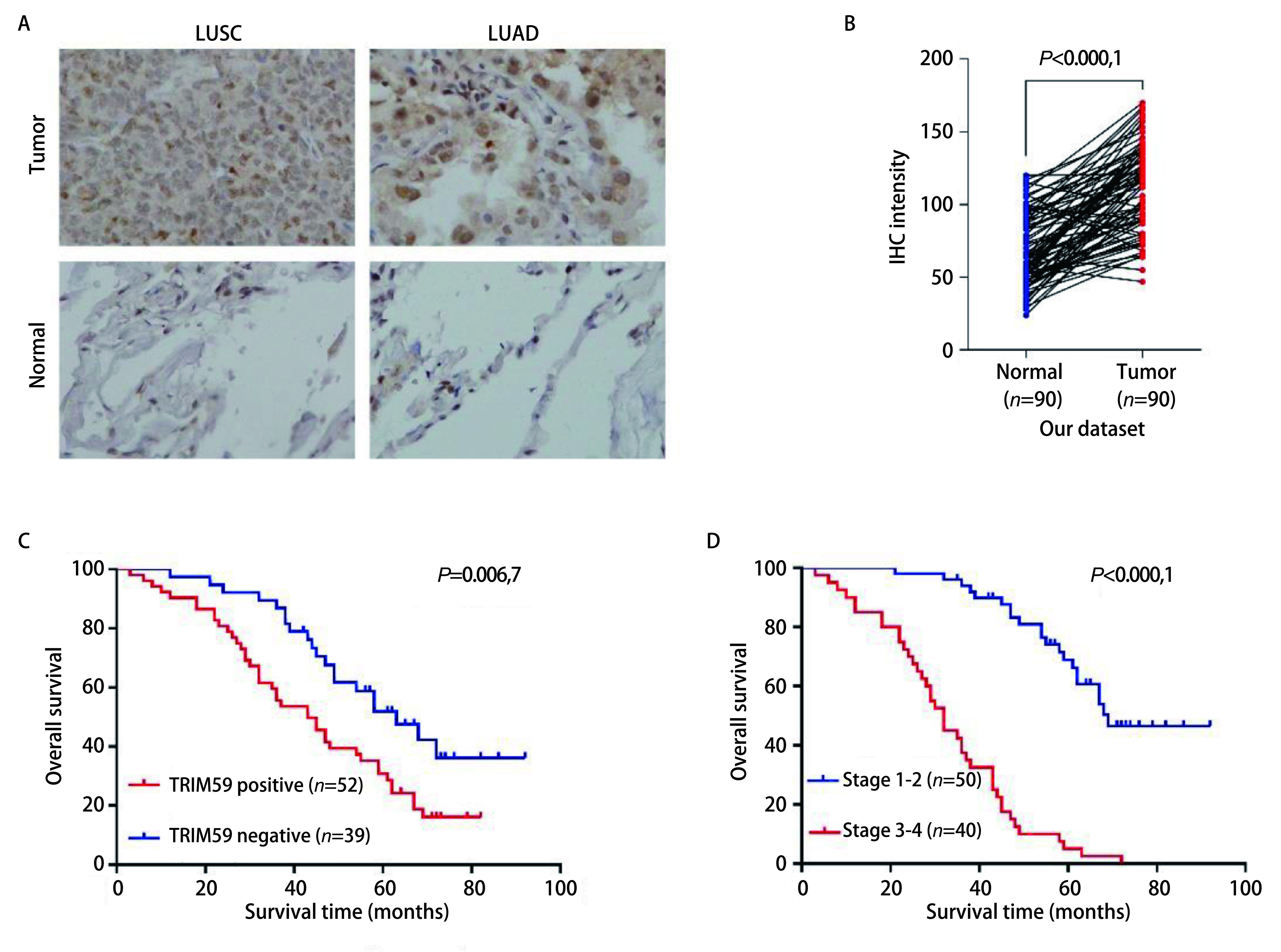
TRIM59蛋白高表达与NSCLC患者预后不良相关。A-B：肺癌组织及癌旁组织TRIM59蛋白的免疫组化结果（LUSC：肺鳞癌；LUAD:肺腺癌）；C：我院90例NSCLC患者的生存分析（*P*=0.006, 7）；D：不同TNM分期患者的生存分析 TRIM59 protein overexpression was associated with poor prognosis in NSCLC. A-B: Immunohistochemical staining of TRIM59 in tumor tissues and adjacent tissues; C: *Kaplan-Meier* survival analysis of NSCLC patients from our hospital (*P*=0.006, 7); D: *Kaplan-Meier* survival analysis of NSCLC patients with different TNM stage. LUSC: lung squamous cell carcinoma; LUAD: lung adenocarcinoma. TNM: tumor-nodemetastasis

**1:** TRIM59蛋白表达与NSCLC患者临床病理特征的关系 Correlation between TRIM59 protein expression and clinicopathological features of patients with NSCLC

Variables	*n*	TRIM59 expression		*P*
		Positive	Negative	
Age (yr)				0.852
< 58	27	16	11	
≥58	63	36	27	
Gender				0.357
Male	47	25	22	
Female	43	27	16	
Histological type				0.528
Squamous cell carcinoma	39	24	15	
Adenocarcinoma	51	28	23	
Tumor differentiation				0.009
Well	52	24	28	
Poor	38	28	10	
TNM stage				0.003
Ⅰ+Ⅱ	50	22	28	
Ⅲ+Ⅳ	40	30	10	
Lymph node metastasis				0.003
No	45	19	26	
Yes	45	33	12	
Tumor size				0.007
< 3 cm	42	18	24	
≥3 cm	48	34	14	

### 2.4 TRIM59蛋白的表达水平与NSCLC患者预后的关系

进一步分析TRIM59蛋白表达水平与非小细胞肺癌患者预后的关系，结果显示TRIM59蛋白的表达水平与患者预后密切相关，TRIM59蛋白高表达的患者预后较差（图 3C）。临床工作中TNM分期是预测患者预后的重要指标，晚期肺癌患者的生存时间明显短于早期肺癌患者（图 3D）。多因素Cox比例风险模型分析显示TNM分期（*P* < 0.001）和TRIM59(*P*=0.005）的表达可作为独立的预测因子来预测患者的预后（表 2）。

**2:** NSCLC患者总生存期的单因素和多因素*Cox*比例风险模型分析 Univariate and multivariate *Cox* regression analysis for overall survival in NSCLC patients

Characteristics	HR (95%CI)	*P*
Univariate analysis		
Age	1.396 (0.788-2.472)	0.253
Gender	1.255 (0.729-2.159)	0.412
Histological type	1.358 (0.818-2.254)	0.237
Tumor differentiation	1.487 (0.895-2.469)	0.125
TNM stage	7.723 (4.390-13.585)	< 0.001
Lymph node metastasis	7.302 (4.044-13.185)	< 0.001
Tumor size	1.569 (0.941-2.616)	0.084
TRIM59 expression	2.052 (1.200-3.507)	0.009
Multivariate analysis		
TNM stage	8.604 (4.520-16.377)	< 0.001
TRIM59 expression	2.306 (1.291-4.117)	0.005

## 3 讨论

肺癌是全球发病和死亡率最高的恶性肿瘤之一，由于局部侵袭、远处转移及耐药等原因，每年因肺癌死亡的患者约占所有癌症死亡患者总数的1/5，居癌症之首^[9]^。现阶段肺癌相关基因的研究一直是国内外学者关注的热点，但迄今为止，肺癌的发生和发展机制尚未完全阐明。前期通过第二代测序技术发现TRIM（Tripartite motif）家族的多个基因（*TRIM25*、*TRIM28*、*TRIM47*、*TRIM59*、*TRIM66*等）在癌组织中表达显著升高，同时我们发现TRIM47可能通过泛素化修饰降解P53和IκB，进而参与P53和核转录因子（nuclear transcription factor-κB, NF-κB）的调控，促进NSCLC的增殖和转移^[10]^。目前关于TRIM59在NSCLC中的表达情况及作用的研究较少。

TRIM家族蛋白是E3泛素连接酶的重要成员^[11]^，越来越多的研究证实TRIM家族成员在肿瘤的发生发展中发挥了重要作用，参与细胞信号传导，调控肿瘤相关分子的表达、化疗耐药等，这为研究TRIM家族蛋白在癌症中的作用机制提供了科学的理论依据^[12, 13]^。TRIM59蛋白是由*TRIM59*基因编码的蛋白质，是TRIM亚家族C-XI的一员，因具有RING结构域而有E3泛素连接酶活性，在许多病理生理过程中发挥重要作用^[14, 15]^。国内学者^[4]^研究发现TRIM59在胃癌组织中高表达，进一步研究发现TRIM59可以与P53结合，促进了P53的泛素化降解，进而促进了癌细胞的增殖和转移。其他学者^[16, 17]^在骨肉瘤和宫颈癌的研究中同样发现TRIM59作为重要的癌基因促进了肿瘤的发生发展。

随着世界各国及科研单位对大数据的重视及财力物力的投入，建立了众多由癌症微阵列数据为主导的数据检索平台，包括TCGA数据库、GEO数据库等。通过挖掘上述公共数据库发表了许多重量级的文章，得到了越来越多研究者的重视^[18]^。本研究中，我们首先从mRNA水平探讨TRIM59在NSCLC中的表达及与预后的关系。前期高通量测序结果显示TRIM59 mRNA在NSCLC组织中表达升高，为了验证这一结果，我们对GEO数据库和TCGA数据库进行挖掘，发现GSE19804数据集、GSE19188数据集、TCGA肺腺癌数据集和TCGA肺鳞癌数据集中肺癌组织TRIM59 mRNA的表达均高于癌旁组织，且随着分期的增高，TRIM59 mRNA的表达量有增高的趋势。通过对GEO数据库和TCGA数据库中的临床数据进行预后分析发现TRIM59 mRNA表达高的肺癌患者，无论是无复发生存期还是总生存期均低于TRIM59 mRNA表达低的肺癌患者。

接下来，我们从蛋白水平探讨TRIM59在NSCLC中的表达及与预后的关系。通过免疫组化方法分析了90例NSCLC患者的存档病理切片，其中TRIM59蛋白阳性表达患者52例，阴性表达患者38例，阳性表达患者的总生存时间较阴性表达患者短。*Cox*多因素回归分析证实TRIM59和TNM分期是预测NSCLC患者预后的独立危险因素。上述结果提示TRIM59可能在NSCLC的发生发展过程中发挥了重要作用，TRIM59的阳性表达可作为预测预后的独立因素，这为深入研究NSCLC的标志物以及靶标治疗提供了一条新思路。但本研究仅从mRNA和蛋白层面分析了TRIM59在NSCLC中的表达及与预后的关系，TRIM59在NSCLC发生发展中的具体作用及作用机制尚需进一步的深入研究。

## Author contributions

Han YD and Jiao WJ conceived and designed the study. Tian HY and Zhang DY performed the experiments. Xu RJ, Qin Y and Lan YL analyzed the data. Han YD provided critical inputs on design, analysis, and interpretation of the study. All the authors had access to the data. All authors read and approved the final manuscript as submitted.
